# Review on radiomic analysis in ^18^F-fluorodeoxyglucose positron emission tomography for prediction of melanoma outcomes

**DOI:** 10.1186/s40644-024-00732-5

**Published:** 2024-07-05

**Authors:** Karim Amrane, Coline Le Meur, Philippe Thuillier, Christian Berthou, Arnaud Uguen, Désirée Deandreis, David Bourhis, Vincent Bourbonne, Ronan Abgral

**Affiliations:** 1 Department of Oncology, Regional Hospital of Morlaix, Morlaix, 29600 France; 2 Lymphocytes B et Autoimmunité, Inserm, UMR1227, Univ Brest, Inserm, LabEx IGO, Brest, France; 3 Department of Radiotherapy, University Hospital of Brest, Brest, France; 4 Department of Endocrinology, University Hospital of Brest, Brest, France; 5 UMR Inserm 1304 GETBO, University of Western Brittany, Brest, IFR 148 France; 6 Department of Hematology, University Hospital of Brest, Brest, France; 7 Department of Pathology, University Hospital of Brest, Brest, France; 8 Department of Nuclear Medicine, Gustave Roussy Institute, University of Paris Saclay, Paris, France; 9 Department of Nuclear Medicine, University Hospital of Brest, Brest, France; 10 Inserm, UMR1101, LaTIM, University of Western Brittany, Brest, France

**Keywords:** Melanoma, Radiomic, FDG-PET, Immune checkpoint inhibition, Immunotherapy, BRAF, NRAS

## Abstract

Over the past decade, several strategies have revolutionized the clinical management of patients with cutaneous melanoma (CM), including immunotherapy and targeted tyrosine kinase inhibitor (TKI)-based therapies. Indeed, immune checkpoint inhibitors (ICIs), alone or in combination, represent the standard of care for patients with advanced disease without an actionable mutation. Notably BRAF combined with MEK inhibitors represent the therapeutic standard for disease disclosing BRAF mutation. At the same time, FDG PET/CT has become part of the routine staging and evaluation of patients with cutaneous melanoma. There is growing interest in using FDG PET/CT measurements to predict response to ICI therapy and/or target therapy. While semiquantitative values such as standardized uptake value (SUV) are limited for predicting outcome, new measures including tumor metabolic volume, total lesion glycolysis and radiomics seem promising as potential imaging biomarkers for nuclear medicine. The aim of this review, prepared by an interdisciplinary group of experts, is to take stock of the current literature on radiomics approaches that could improve outcomes in CM.

## Introduction

Cutaneous melanoma (CM) results from genetic mutations in melanocytes and accounts for approximately 1% of all malignant skin tumors.

It mainly affects the Caucasian population of both sexes [1], over 75 years of age [2] and is the 15th most common cancer worldwide. Its incidence has increased more rapidly than other cancers in recent decades [3] and varies considerably between countries due to differences in UV exposure and skin phototype [4]. The 3 countries with the highest incidence rate of CM are in Europe (Denmark, Sweden, and the Netherlands). In case of distant metastases, the prognosis is very poor; with a 5-year survival rate of 99.4% (95% CI: 98.9–100.0) for stage I versus 23.0% (95% CI: 10.3–51.4) for stage IV [3] according to the AJCC melanoma staging system [5].

Several common prognostic factors have been highlighted in the literature, including age, gender, nutritional status, performance status (PS), tumor size, Breslow thickness, mitotic rate and ulceration, lymph node involvement, distant metastasis, anatomical site (axial localization), surgery of the primary lesion, time-to-progression after first-line therapy, and biomarkers (BRAF, NRAS) status [4, 6].

Immunotherapy, such as immune checkpoint inhibitors (ICIs) that block CTLA-4 (e.g., ipilimumab) [7], PD-1 (e.g., nivolumab, pembrolizumab) [8–10] and LAG3 (e.g., relatimab) [11], have demonstrated objective tumor regression in patients with advanced melanoma, using the activation of the immune system to generate an anti-tumor response.

The 5-year survival rate for CM with ipilimumab is estimated to be between 12.3% and 16.5%, with a prolonged response time [12], while nivolumab has a 5-year survival rate of 39% with a response rate of 42% [13]. On the other hand, the combination of anti-PD1 with anti-CTLA4 (ipilimumab) or anti-LAG3 (relatimab) has shown better results than monotherapy. Indeed, the nivolumab-ipilimumab combination showed a 5-year survival rate of 52%, and a response rate of 58%; while data on the nivolumab-relatimab combination are still immature. However, the 12-month progression-free survival (PFS) rate is 47.7% [14, 15]. Tyrosine kinase inhibitors (TKIs), which target kinases in the mitogen-activated protein kinase (MAPK) pathway, are approved for metastatic BRAF-mutant CM, and prolong patient survival. Response to these therapies is limited by drug resistance and is less durable than with ICIs. Early clinical trials have shown that BRAF inhibitors (BRAFi) (vemurafenib, dabrafenib and encorafenib) improve the median PFS compared with chemotherapy in patients with BRAF-activating somatic mutations [16, 17]. However, the efficacy of BRAFi as monotherapy is limited due to the development of drug resistance, resulting in little difference in long-term survival rates compared with chemotherapy [18, 19]. Targeted therapies combining BRAFi and MEKi (dabrafenib plus trametinib, vemurafenib plus cobimetinib, encorafenib plus binimetinib), which act downstream of BRAF in the MAPK pathway, have been tested to enhance inhibition of the MAPK pathway and delay acquired resistance to BRAFi,. Combination therapy improved median PFS compared with BRAFi monotherapy [20, 21].

Although most patients respond immediately to BRAFi-MEKi combination therapy (objective response rate (ORR) of around 70%), long-term PFS after treatment affects only a subset of patients (5-year PFS rates range from 14–22.9% [22–24]). Whereas ICI combination therapy has a slightly lower ORR (around 60%) but shows a more durable response in BRAF-mutant CM (36% 5-year PFS rate of 36% and 6.5-year PFS rate of 34% with nivolumab plus ipilimumab [15, 25]). Finally, as suggested by phase II studies, combinations with ICI have been evaluated to enhance MAPK inhibition and delay acquired resistance to BRAFi MEKi [26]. However, only one trial was able to compare the ICI-BRAFi-MEKi combination against BRAFi-MEKi, but did not show superiority in patients with advanced BRAFV600-mutated melanoma [27].

Despite the paradigm shift brought about by ICIs (prolonged survival and good tolerance [28–31]), 40–65% of metastatic melanomas do not respond to mono- or combo-ICIs and more than 43% of patients develop secondary resistance after an initial response after 3 years of treatment [28].

Pre-therapeutic ^18^F-fluorodeoxyglucose positron-emission tomography (FDG-PET/CT) is currently indicated for the staging of locally advanced CM [32]. In addition, several studies have shown that tumor uptake, assessed by different standard quantitative static (SUV = standardized uptake value), and volumetric (MTV = metabolic tumor volume, TLG = total lesion glycolysis) parameters, is a prognostic factor for survival in various solid cancers, including CM [33–35]. However, no clear cut-off value for the selection of patients with poor prognosis has been validated in practice.

More recently, radiomic approach has been developed to characterize tumor heterogeneity by extracting textural features (TFs) from different imaging modalities, including PET. CM also exhibits high biological heterogeneity with hypoxic areas, necrotic regions, zones of high cellular proliferation and intra-tumoral angiogenic heterogeneity [36, 37]. SUV, MTV and TLG can by definition be considered as first-order radiomic features, but do not provide information concerning on the spatial distribution of voxel values. Therefore, second and higher order parameters allow the modelling of this spatial relationship with numerous TFs [38]. This image-based approach may lead to a more personalized therapy in this particular poor prognosis cancer entity, where intensification of treatment could lead to improved clinical outcomes. Given the narrow therapeutic window of such approaches, efficient prognostic and predictive tools are needed. Texture analysis of the tumor on pre-therapeutic FDG-PET/CT may also be useful, although there are currently many pitfalls in radiomics in terms of reproducibility and calibration of PET machines. Nevertheless, the development of artificial intelligence and deep learning brings a new insights and could improve the robustness of this technique [39].

Given the significant potential of radiomics, we first present an overview of the basics of radiomics, then the potential contribution of radiomics to prognosis, the predictive approach to mutation status and, finally, we discuss the methodological aspect and its limitations before turning to strategies for harmonizing practices.

## Basics of radiomics

Radiomics is a process based on the extraction of large sets of quantitative features (e.g., volume, shape, intensity, texture, etc.) from medical images, using a high-throughput computer system [40]. Radiomics aims to extract the full power of images in order to improve clinical decision support by integrating image-derived information into the clinic.

**Fig. 1:**
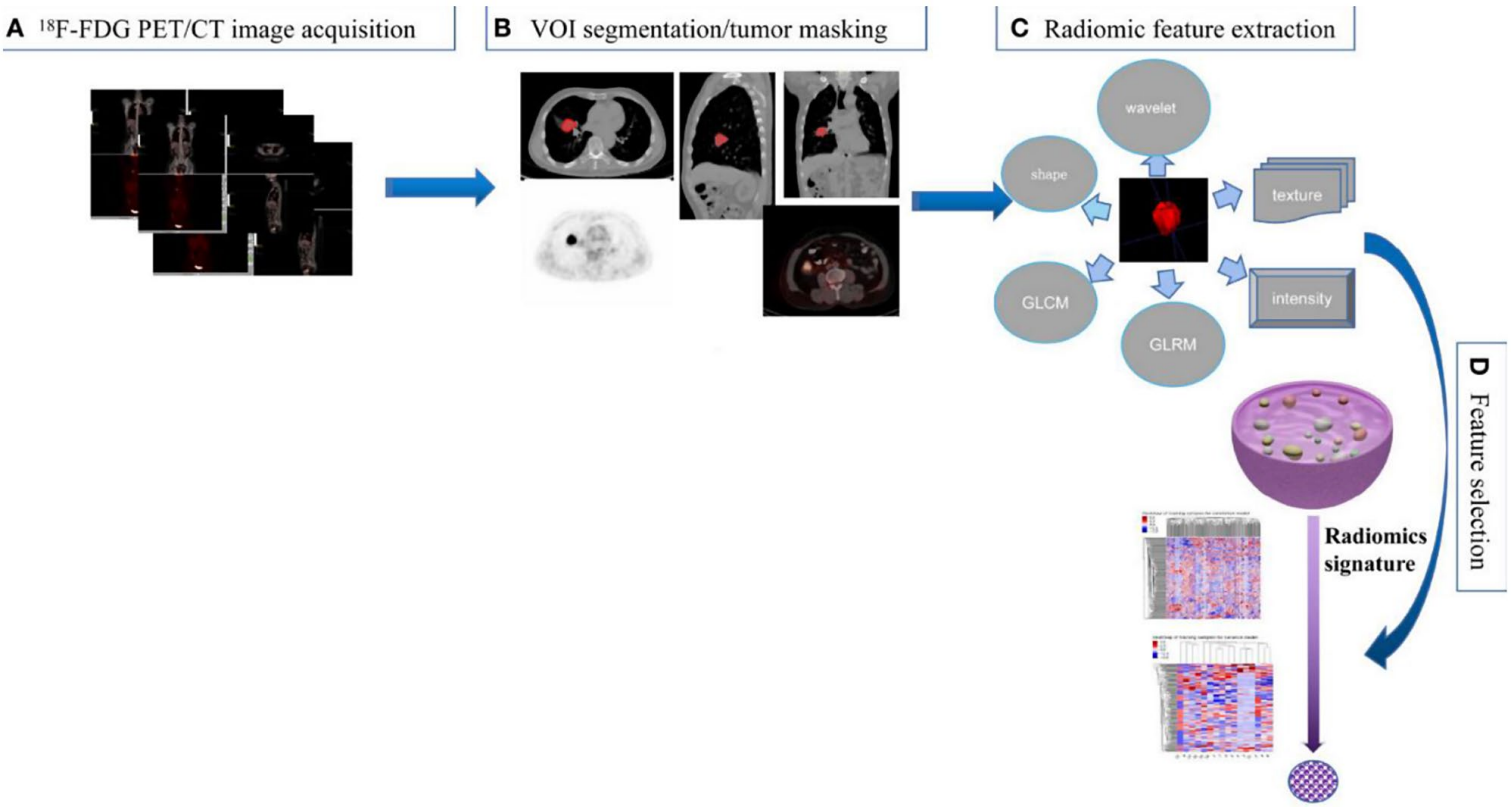
The radiomics workflow consists of several steps: (**a**) image acquisition, (**b**) VOI segmentation, (**c**) radiomic feature extraction and (**d**) feature selection [41]. Is taken from the article Radiomics in Nuclear Medicine Applied to Radiation Therapy: Methods, Pitfalls and Challenges. Sylvain Reuzé,Antoine Schernberg, Fanny Orlhac, Roger Sun, Cyrus Chargari, Laurent Dercle, Eric Deutsch, Irène Buvat, Charlotte Robert [38]. License number authorizing re-use obtained from the publisher ELSEVIER / 5,660,690,860,547

The radiomics workflow consists of several steps: (a) image acquisition, (b) VOI segmentation, (c) radiomic feature extraction and (d) feature selection (Fig. 1). [41]

**Fig. 2:**
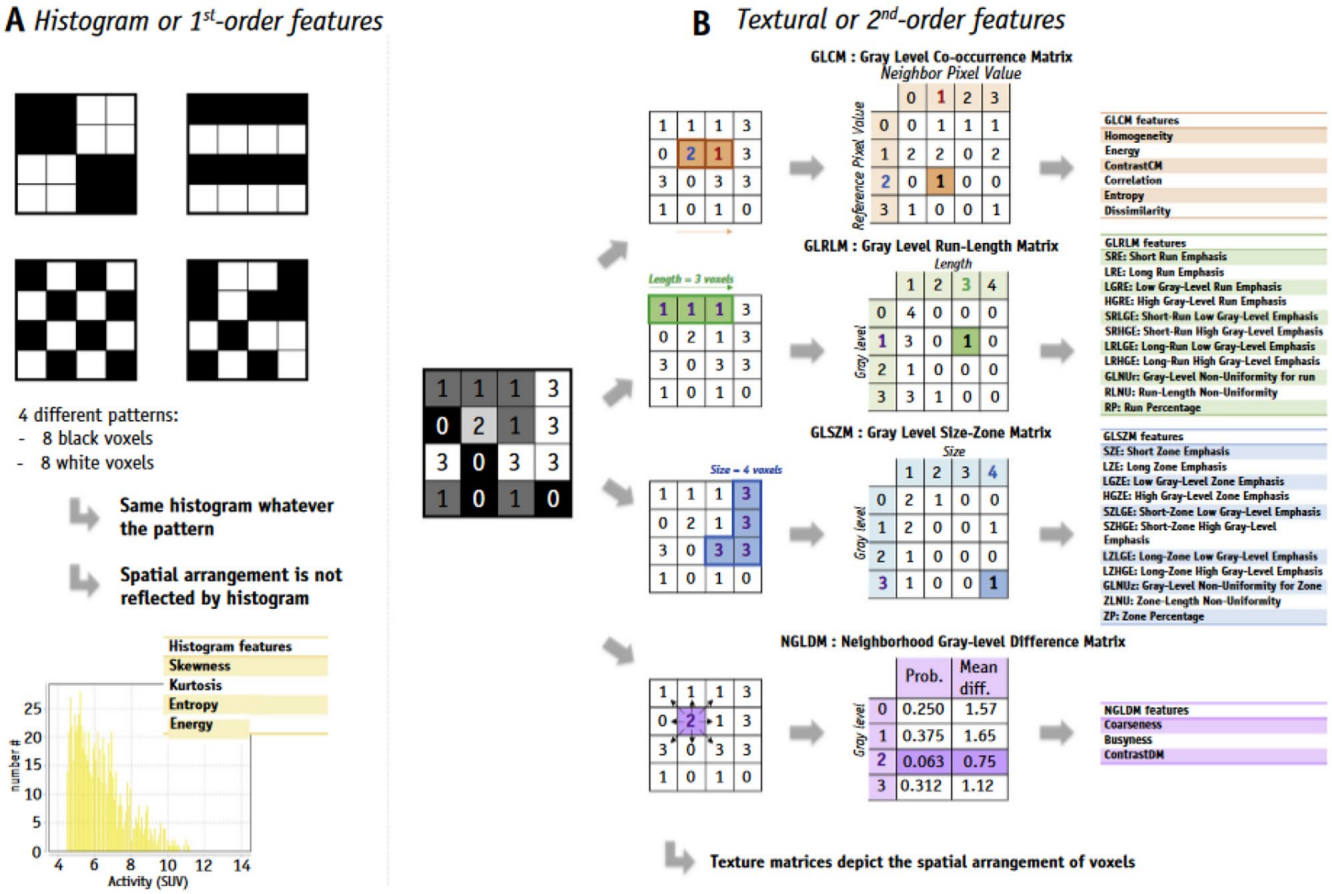
Common texture matrices and derived texture indexes (Abbreviations: defined in the main text) [38]. Is from the article PET/CT Radiomic Features: A Potential Biomarker for EGFR Mutation Status and Survival Outcome Prediction in NSCLC Patients Treated With TKIs Published in Front. Oncol, 21 June 2022 s. Thoracic Oncology Volume 12–2022 | 10.3389/fonc.2022.894323. Copyright © 2022 Yang, Xu, Li, Wang, Peng, Zhang, Wu, Chu, Wang, Meng and Zhang [41]

Several types of data can be extracted from PET imaging. Qualitative semantic features are commonly used in the clinical lexicon to describe lesions [42]. They have different levels of complexity, expressing firstly the properties of the lesion shape and voxel intensity histogram, and then those of the spatial arrangement of intensity values at the voxel level (texture). They can be extracted directly from the images or after applying various filters or transformations (e.g., the wavelet transform) (Fig. 2– Table 1) [38].

**Table 1:**
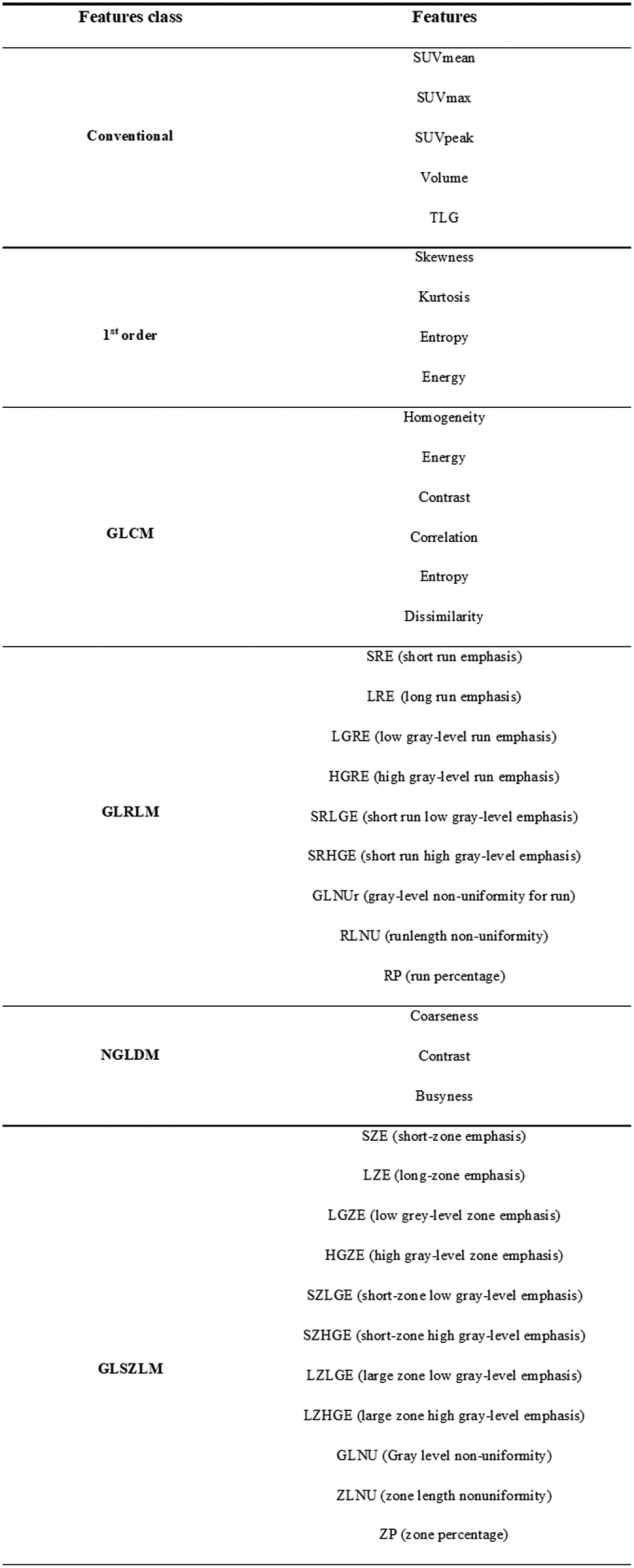
Groups of features

First-order features, or histograms, describe the distribution of individual voxel values or gray-scale intensities within a VOI, independent of spatial relationships. Histogram features are therefore not TFs, but describe the shape of the intensity histogram, such as kurtosis or flattening. Different spatial arrangements of gray levels, corresponding to the same histogram, can therefore give rise to identical index values. From the histogram, it is possible to calculate conventional parameters such as maximum (SUVmax), mean (SUVmean), peak (SUV peak defined as highest mean value in a spherical VOI of 1 cc), metabolic tumor volume (MTV), total lesion glycolysis (TLG = MTV x SUVmean), and skewness, kurtosis, energy and entropy.

Second- and third-order statistical features include so-called TFs [43, 44], which are obtained by calculating the statistical relationships between neighboring voxels [38]. They provide a measure of the spatial arrangement of voxel intensities, and thus of intra-lesional heterogeneity. These features can be derived from the Gray Level Co-occurrence Matrix (GLCM), which quantifies the occurrence of voxels of the same intensity at a given distance along a fixed direction, or the Gray Level Run Length Matrix (GLRLM), which quantifies successive voxels of the same intensity along fixed directions. The Gray Level Size-Zone Matrix (GLSZM) measures the number of neighboring voxels of the same intensity, for each intensity value. The Gray-Level Difference Matrix (NGDLM) measures the difference in intensity between neighboring voxels.

Higher-order statistical features are obtained using statistical methods after applying filters or mathematical transformations to the images, for example to identify repetitive or non-repetitive patterns, remove noise or highlight details. These include fractal analysis, Minkowski functions, wavelet transforms and Laplacian transforms of Gaussian-filtered images, which can extract areas of increasingly coarse texture patterns.

### Indications of FDG PET/CT

FDG PET/CT plays a clear role in the diagnosis of advanced melanoma, and well-designed studies will determine whether it is appropriate to include FDG PET/CT in the management and follow-up of patients with metastatic CM. FDG PET is recommended for AJCC stage III (regional lymph nodes) and IV (systemic) melanoma for staging, restaging and follow-up treatment, with a significant implications on management [32]. For primary staging of stage IV melanoma, overall estimates of the sensitivity of FDG PET/CT over CT were 80% versus 51%, and for the specificity 87% versus 69%. However, although FDG PET/CT is an excellent tool, it is not currently recommended for routine follow-up of asymptomatic patients with stage I to IIIA disease or for prognostic evaluation of CM [32]. FDG-PET can detect clinically relevant immune-related adverse events prior to clinical diagnosis, including endocrinopathies and enterocolitis [45].

Finally, it may help to understand the distinction between the categories of pseudoprogression, hyperprogression, dissociated/mixed response and sustained response in the interpretation of FDG-PET, several studies are underway [46].

### Methods

#### Search strategy

Two authors (K.A. and C.L.M.) together performed a computerized bibliographic search of the Medline databases in September 2023. The following search terms (“Melanoma”) AND (“PET” OR “positron emission tomography”) AND (“radiomics” OR “radiomic” OR “texture” OR “textural”) AND (“Immune checkpoint-inhibitor” OR “Immunotherapy”) were used according to a free text protocol that applied only “Humans” and “English language” filters without time-period restriction.

#### Criteria

Records were considered relevant to this review if they included melanoma FDG-PET/CT textural analysis and its prognosis impact, predictive mutational status approach with radiomic, methodological aspect of radiomic studies and practice harmonization. Case reports, editorials, letters, and meeting abstracts were excluded during the review process.

Studies were selected according to the following criteria: (i) only studies that included patients who had been treated for melanoma; (ii) only studies with patient numbers ≥ 50; (iii) only studies that included exclusive pretherapeutic FDG-PET; iiii) only studies that included textural features analysis.

#### Review process

After duplicates were removed, two authors (K.A. and C.L.M.) completed an independent review of 66 abstracts to ultimately select 10 studies for separate full-text evaluation. Any discrepancies in study inclusion were resolved by consulting the supervisor of the review process (V.B. and R.A.).

## Prognostic approach

Radiomic application hold promise for treatment personalization. Several parameters can be extracted and combined, including standard descriptors (e.g., size, morphology, TNM stage (tumor, lymph nodes, metastasis)), qualitative, semi-quantitative physiological parameters (e.g., contrast enhancement, scattering characteristics, tracer uptake), and additional agnostic features that are otherwise “invisible” using bioinformatic approaches. Of these features, texture-based features have been the most widely studied, particularly in FDG-PET/CT [47] and several prognostic approaches have been investigated.

Ito et al. analyzed the prognostic value of pre-therapeutic metabolic FDG-PET/CT parameters in a retrospective study of 142 patients with only advanced CM who received second-line ipilimumab after chemotherapy. As criteria of tumor metabolic activity, the authors determined the SUV corrected for lean body mass (SUL) of all lesions and to assess tumor burden, they defined MTV as the volume bounded by a 42% SUVmax threshold of tumor lesions. Occasional manual adjustments were made when the defined volume exceeded the limits of the lesions seen on CT. Total body (TB) MTV and TLG, defined as the sum of MTV and TLG of each hypermetabolic metastatic lesion, were calculated for each patient. TB-MTV and TB-TLG were significantly associated with overall survival (OS) unlike SULmax (*P* = 0.60) and SULpeak (*P* = 0.056). The median OS for patients with TB-MTV above the median was 10.84 months (95% CI, 5.88–5.81 months) as compared to 26.09 months (95% CI, 3.02–49.15 months) for patients with a TB-MTV below the median (*P* = 0.002, HR = 1.9). The corresponding 1-, 2- and 3-year survival rates were 47% vs. 72%, 28% vs. 51%, and 21% vs. 46% in these two subgroups, respectively. The prognostic value of TB-TLG and TB-MTV was equivalent. The median OS for patients with TB-TLG above the median was 10.84 months (95%CI: 5.50–16.19 months) as compared to 22.34 months (95%CI: 9.36–35.32 months) for patients with TB-TLG below the median. Differences in survival between patients with a sum of SULpeak above and below the median were not significant, but a trend was observed (*P* = 0.056, HR = 1.48). Image analysis was performed by a single experienced physician, certified in radiology and nuclear medicine, who had access to the patient’s clinical data. Nevertheless, image interpretations were confirmed by another physician specialized in nuclear medicine, and doubtful results were resolved by consensus between the two investigators. Although this approach is not ideal, it does enable a certain quality of analysis to be maintained [48]. In this study, several FDG-PET/CT from the same manufacturer were used. A cross-calibration between the dose calibrator and the FDG-PET/CT was performed every month, in order to homogenize the results obtained, which is a guarantee of quality. This study had a selection bias because the population, although homogeneous and including brain metastasis were admitted, is not representative of the population eligible for immunotherapy, and the patients had previously received chemotherapy, which may bias the results under immunotherapy. Finally, there was no validation on an external cohort, which would provide greater robustness to the results [49].

In a retrospective single-center study, the analysis of volumetric parameters on initial PET prior to ICI induction was investigated in 56 patients with unresectable cutaneous (*n* = 32) or mucosal (*n* = 24) melanomas. Parameters extracted from tumor tissue were SUVmax, SUVmean, TB-MTV and from lymphoid tissue were bone marrow-to-liver ratio (BLR) and spleen-to-liver ratio (SLR). For the 2 matched melanoma subtypes (mucosal melanoma; cutaneous melanoma), (ORRs) were 33% and 42%, disease control rates (DCRs) were 56% and 69%, median follow-up were 25.0 and 28.9 months, median PFS were 4.7 and 10.7 months, and median OS were 23.9 and 28.3 months, respectively. For the CM subtype, increasing TB-MTV above 16.3 ml and BLR above 0.76 were independently associated with a shorter OS (both *P* < 0.001) and a shorter PFS (*P* = 0.01 and *P* = 0.04, respectively) [50]. In this study, a single GE Discovery 690 FDG-PET/CT was used to ensure homogeneity of results and quality of analysis and findings. A pair of expert radiologists and nuclear medicine physicians analyzed the FDG PET/CT scans and the contrast-enhanced CT scans and segmented the lesions. The readers were blinded and, unlike in the study conducted by Ito et al., had no knowledge of the patients’ medical or pathological history, imaging characteristics or clinical outcome,. [48]. This approach is of high methodological quality. However, it has a number of limitations: firstly, it is a heterogeneous population consisting of two melanoma subtypes with different prognosis and response to ICI [51, 52]. Secondly, the population is not homogeneous in terms of the treatments received, since in addition to the difference in the regimens administered in the first line, either anti-CTLA-4 or anti-PD1, there is also the fact of analyzing patients who received ICI in the second line. These elements help to explain the results, in addition to the small number of patients, in favor of a weak robustness, as the authors report. Finally, there was no validation in an external cohort.

In a meta-analysis of 24 studies analyzing the prognostic value of FDG-PET/CT in patients with melanoma under ICI, 13 studies used baseline scans. First-order radiomic variables, e.g. SUV, SUL, MTV, TLG, were the most commonly used. The pooled hazard ratios (HR) of MTV, SLR, SUV/SULmax, SUV/SULpeak, and TLG for OS were 1.777 (95% CI: 1.389–2.275, *P* < 0. 001), 3.425 (95% CI: 1.707–6.869, *P* = 0.001), 0.941 (95% CI: 0.599–1.477, *P* = 0.791), 1.704 (95% CI: 1.253–2.316, *P* = 0.016), and 1.755 (95% CI: 1.315–2.342, *P* < 0.001), respectively. The conventional and modified response assessment criteria had a pooled sensitivity of 64% (95% CI: 46–79%) and 94% (95% CI: 81–99%) and a pooled specificity of 80% (95% CI: 59–93%) and 84% (95% CI: 64–95%), respectively [53].

Furthermore, in a retrospective series of 92 patients with advanced melanoma including 27% with BRAF mutation treated with anti-PD1 as first-line therapy, Nakamoto et al. also explored the association between FDG uptake by immune-mediating tissues, such as the bone marrow (BM) and spleen, and poor cancer outcomes. FDG/PET-CT scans were performed on different machines, which could lead to variability in SUV measurements of MTV. However, BM and spleen metabolism estimates were corrected for liver background as ratio (BLR and SLR, respectively), which harmonized the PET characteristics. MTV and TLG were calculated using an absolute threshold of SUV = 2.5. Liver and spleen SUVmean were measured by drawing a spherical VOI in the center of a non-disease area of the right hepatic lobe (3 cm) and spleen (2 cm), respectively. For the BM, 1.5-cm spherical VOIs were placed in the center of a non-diseased L1–L4 vertebral bodies, and an average SUVmean was calculated for the lumbar vertebral bodies. After a median follow-up of 18.2 months, 57.6% (53/92) patients had disease progression, and 34.8% (32/90) of them died. Median PFS and OS were 11.6 months (95% CI, 7.1–28.3 months) and more than 60 months, respectively. Both BLR and BRAF mutation were independent prognostic factors for PFS (*P* = 0.017 and 0.018, respectively); and BLR, BRAF mutation, and lactate dehydrogenase (LDH) elevation were independent prognostic factors for OS (*P* = 0.011, 0.0078, and 0.013, respectively). The median PFS of the high BLR (> 0.78) group was 8.6 months (95% CI: 3.0–42.5 months), significantly shorter than that of the low BLR group at 28.3 months (95% CI: 7.7–54.9 months, *P* = 0.027). Similarly, the median OS of the high BLR group was 28.0 months (95% CI: 17.2–28.7 months), significantly shorter than that of the low BLR group > 60 months (*P* = 0.019) [54]. In this study, several FDG-PET/CT scanners from the same manufacturer were used as in the ITO study [48]. The use of different PET/CT scanners may have led to variability in SUV MTV measurements. However, the estimation of BM metabolism was assessed by normalizing the values to the liver background, thus harmonizing the PET characteristics and generalizing the model studied. Nevertheless, the authors did not specify the analysis modalities, which may have an impact on the quality of the study, unlike the previously cited studies [48, 50]. On the other hand, this was a generally homogeneous population, as brain metastases and BRAF-mutated patients were included, and all patients received first-line anti-PD1 therapy, although almost 12% received an anti-PD1-based combination. The retrospective nature of the study also induces selection bias. also note the absence of external validation, as in previous reported studies [48, 50].

In another retrospective study, two experienced nuclear medicine specialists performed a visual and semi-quantitative assessment of all lesions in 51 patients with CM who underwent FDG-PET/CT. Data were collected on the number of lesions in total and according to 5 body regions (head and neck, chest, abdomen and pelvis, skeleton and extremities, with bone lesions always counted in the skeleton). Usual metabolic parameters were collected (SULmax, SULpeak, SULmean, TB-MTV, TB-TLG) and tumor-to-background ratios (TBRs) calculated using mean activity in the liver (TLR), blood-pool (TBR), and muscle (TMR) at baseline, 3 months and 6 months after treatment initiation. The OS rates at 3 and 5 years were 49% and 43.1%, respectively. From baseline FDG-PET/CT, only the SULmax and SULpeak, as well as most TBRs, were predictive of 3- and 5-year OS rates. From 3 to 6 months FDG-PET/CT, TB-MTV, TB-TLG and most TBRs were predictive of survival. Changes in MTV, TLG, and most TBRs (delta values) between baseline and the 3- and 6-month follow-up scans had prognostic value. In the multivariate analysis, of all the parameters analyzed, the most predictive of OS were extracted from the 3-month follow-up scan. TBR was the best reported predictive factor (cut-off value of 2.15, sensitivity of 88.5%, and specificity of 68.0% for 3-year survival) [55]. Two different FDG-PET/CT systems were used in this study. All studies were reanalyzed using Philips IntelliSpace Portal software, version 10.1. The authors also point out that their center is accredited by the European Association of Nuclear Medicine Forschungs GmbH (EARL) and that all acquisitions were performed according to EARL criteria (in order to maintain compatibility between data obtained from the two scanners), a quality guarantee not found in the previous reports cited. The authors point out that, despite the use of EARL standards to obtain the most accurate quantification possible, certain biases remain unavoidable. The reproducibility of TLG and MTV measurements had not been systematically studied in all patients. To eliminate the subjectivity associated with selecting a specific number of lesions to obtain reproducible quantitative results, the authors measured the sum of PET volumetric metabolic parameters for all lesions, specified as wMTV and wTLG. As in the study by Ito and Seban [50, 56], all images were analyzed by two experienced nuclear medicine specialists, who made a qualitative (visual) and semi-quantitative assessment of all lesions. The population is not sufficiently described to assess its homogeneity, in fact, there is no precision regarding mutation status or the presence of brain metastases. Nevertheless, all patients had received ICI as fiRST-line treatment, but with different regimens. The majority of patients (62.7%) received anti-CTLA4, followed by anti-PD1 (31.4%) and a combination of anti-PD1 and anti-CTLA4 (5.9%). This treatment heterogeneity between anti-PD1 and CTLA4 may have influenced the results reported by the authors. In addition to the retrospective nature and relatively small sample size, which are sources of selection bias. It is important to note that there was no assessment of response rate and a lack of external validation.

Annovazzi et al. reported on a retrospective study of 57 patients with advanced BRAF-mutated melanoma treated with BRAF ± MEK inhibitors as first-line therapy. The authors analyzed the prognostic value of pre-therapeutic metabolic parameters and investigated the association between FDG uptake by immune-mediating tissues, such as BM and spleen, and poor cancer outcome. Thirty-four patients were classified as responders (metabolic partial/complete response [PR/CR]) and 23 as non-responders (metabolic progressive/stable disease [PD/SD]). Baseline MTV > 56 ml was independently associated with a shorter PFS. Patients who achieved a CR were associated with longer PFS compared to those who achieved a PR (median of 42.9 vs. 8.8 months respectively, *P* = 0.009). Disease progression occurred in new disease sites in 87.5% of CR, 7.1% of PR and 34.8% of PD/SD (*P* < 0.001). High baseline TB-MTV and lack of treatment response were independent prognostic factors for OS, stratifying patients into 3 different prognostic classes (median OS 6.7, 18.3 and 102.2 months, respectively) [57]. Only one FDG-PET/CT scanner was used in this study which guarantees the quality for the analysis of the results. The images were analyzed by two nuclear medicine physicians, who were familiar with the clinical data but had no knowledge of the clinical outcome of the patients. The evaluation criteria were the same as for the Ito et al. study [48]. Although the population was relatively small, the study focused exclusively on BRAF-mutated patients treated with the BRAFi+/- MEKi combination, which is unprecedented in the literature. Although the population was drawn from a retrospective collection from a single center, it remains globally homogeneous, with cutaneous melanoma including patients with brain metastases. The patients included had received different treatment regimens including BRAFi as monotherapy (vemurafenib or dabrafenib) or in combination with MEKi (cobimetinib or trametinib, respectively). The retrospective design of the study may had led to selection bias. Another possible limitation of the study is that 17.5% of patients had received systemic therapy prior to BRAFi/MEKi targeted therapy.

In a recent single-center retrospective Australian study of 106 patients with advanced melanoma, who received the combination of ipilimumab and nivolumab, the authors also assessed the prognostic value of first-order parameters from pre-therapy FDG-PET/CT. For each patient, TB-MTV was automatically measured on FDG-PET/CT in the entire field of view (vertex to mid-thigh) using the PERCIST liver SUV threshold (1,5 x mean liver SUV in a 3 cm spherical VOI on the right lobe + 2 SD) [58]. The median TB-MTV and TB-TLG were estimated to be 43 mL and 359 g, respectively. The 12-month survival rate was significantly lower in patients with TB-MTV and TB-TLG higher than the median values (43% [95% CI: 32, 58] vs. 66% [95% CI: 55, 79], *P* < 0.001 and 43% [95% CI: 32, 57] vs. 67% (95% CI: 56, 80], *P <* 0.001, respectively). Similarly, the subgroups of patients with high TB-MTV or TB-TLG had a higher risk of death, with HRs of 1.9 (95% CI: 1.2, 3.0; *P* = 0.007) and 2.0 (95% CI: 1.2, 3.1; *P* = 0.004), respectively [59]. Iravani et al. had used several FDG PET/CT scanners which were cross-calibrated every 3 months, in order to homogenize the results obtained, which is a guarantee of quality. The analysis procedure is qualitative, even though it relied on a single experienced nuclear medicine physician who was blinded to clinical information and patient outcomes. Although the population was large, it was not homogeneous. The authors did not specify the exclusive cutaneous nature of the melanoma, and even if all patients had received a dose of ipilimumab and nivolumab, only 46.2% had received it as first-line treatment, while the other patients had received it as subsequent-line treatment. The lack of external validation reduces the robustness of the results obtained.

Finally, in a retrospective two-center study of 56 patients with metastatic BRAF wild-type melanoma who underwent initial FDG-PET/CT prior to ICI, the primary objective was to predict prognosis based on radiomic parameters. A semi-automated lesion segmentation method was used, based on a relative SUVmax threshold of 40%. Texture analysis was performed in the VOI of the lesion with the highest FDG uptake. Before calculating the TFs, the voxel intensities of these VOIs were resampled using 64 discrete values ranging from 0 to 32 SUV units. Only one TF, Long Zone Emphasis (LZE), was reported to be significantly correlated with OS (LZE cut-off value = − 437) in their series. And both total MTV and LZE were independent prognostic factors for OS. Therefore, the population was stratified into 3 risk categories: low risk if TB-MTV ≤ 5.6 ml and LZE ≤ -437 (*n* = 23; 41%), intermediate risk if TB-MTV > 5.6 ml or LZE > -437 (*n* = 19, 34%), and high risk if TB-MTV > 5.6 ml and LZE > -437 (*n* = 14; 25%). Survival rates were respectively 91.1% (95% CI: 80–100) for the low-risk group, 56.1% (95% CI: 37.1–85) for the intermediate-risk group, and 19% (95% CI: 0.06–60.2) for the high-risk group. The HRs for OS were 0.11 for the low-risk group (95% CI: 0.025–0.46, *P* = 0.0028), 1.2 for the intermediate-risk group (95% CI: 0.48–2.8, *P* = 0.74) and 5.9 for the high-risk group (95% CI : 2.5–14, *P* < 0.0001) [60] (Table 2). The PET/CT scans were acquired with different systems. As this was a retrospective study, no harmonization was performed prior to data collection. However, the ComBat post-reconstruction harmonization method was used to combine the conventional and textural characteristics of the four different PET/CT scanners, 22,23. After ComBat harmonization, a selection of features was used to shrink the model to reduce overfitting and co-variable correlation. The bidirectional correlation of features was assessed using Spearman’s rank correlation coefficient, and those with correlation coefficients greater than 0.8 were removed. The use of the ComBat procedure adds considerable robustness to the study methodology [61].

**Table 2:** Summary of studies evaluating the prognostic value of pre-therapy FDG-PET/CT in metastatic melanoma

Author Number of patients	PET Time point	Therapy	Features assessed	Outcomes	Findings	PET/CT Scanners	Externe validation	Harmonization
Ito et al [48] *n* = 142	Pre-therapeutic	Ipilimumab (second line)	MTV, TLG, SUL_max_, SUL_peak_	Survival prognosis	TB-MTV and TB-TLG predicted survival	GE Discovery Series (VCT, ST, STE, 600 and 690),	No	A cross-calibration between the dose calibrator and the FDG-PET/CT was carried out every month
Seban et al [50] *n* = 56	Pre-therapeutic	Ipilimumab Pembrolziumab	SUV_max_, SUV_mean_, TB-MTV, BLR, SLR	Survival prognosis	TB-MTV > 16.3 ml and BLR > 0.76 was independently associated with a shorter OS (both *P* < 0.001) and a shorter PFS (*P* = 0.01 and *P* = 0.04, respectively)	GE Discovery 690 FDG-PET/CT	No	NA
Nakamoto et al [54] *n* = 92	Pre-therapeutic	Pembrolziumab Nivolumab	SUV_max_, MTV, BLR	Survival prognosis	BLR > 0.78 was independently associated with a shorter PFS and OS (*P* = 0.017 and *P* = 0.011, respectively)	GE Discovery 600, 690, 710 or MI (GE Healthcare),	No	Normalization to hepatic background
Schweighofer-Zwink et al [55] *n* = 51	Pre-therapeutic	Ipilimumab Pembrolziumab Nivolumab	SUL_max_, SUL_peak_, SUL_mean_, TB-MTV, TB-TLG, TBR, TMR	Survival prognosis	TBR was independently associated with survival (cutoff value of 2.15, sensitivity of 88.5%, and specificity of 68.0% for 3-year survival)	Phillips TF Ingenuity and Siemens Biograph mCT scanners	No	Acquisitions were carried out on the basis of EARL criteria (in order to maintain compatibility between data obtained from the two scanners), Use of Philips IntelliSpace Portal software, version 10.1.
Annovazzi et al [57] *n* = 57	Pre-therapeutic	BRAFi MEKi	TB-MTV	Survival prognosis	TB-MTV > 56 ml was independently associated with a shorter PFS and OS	Siemens Biograph16 (Siemens Healthineers),	No	NA
Iravani et al [59] *n* = 106	Pre-therapeutic	Ipilimumab-Nivolumab combination	TB-MTV TB-TLG	Survival prognosis	TB-MTV > 42 ml was independently associated with a shorter PFS and OS	FDG PET/CT scanners, including Biograph 16(Siemens Healthineers), GE 690 or GE 710 Discovery (GE Healthcare) scanners	No	Cross-calibration every 3 months
Flaus et al. [60] *n* = 56	Pre-therapeutic	Pembrolziumab Nivolumab	41 features including: First order statistics, GLCM, GLRLM, GLSZM, NGLDM	Survival prognosis Therapeutic response	Both TB-MTV > 5.6 ml and LZE >-437were independently associated with a shorter OS (*P* < 0.0001)	Biograph mCT Flow 20 (Siemens Healthcare) and Biograph 6 HI-REZ (Siemens Healthcare) Biograph Horizon 16 (Siemens Healthcare) and Discovery 690 (General Electrics Healthcare)	No	ComBat procedure

*Abbreviations* GLCM = gray level co-occurrence matrices; GLRLM = gray-level run length matrix; GLSZM = gray level size zone matrix; NGLDM = neighborhood gray-level different matrix; LZE = long zone emphasis; SUV = standardized uptake value; SUL = SUV corrected by lean body mass; TB = total body; MTV = metabolic tumor volume; TLG = total lesion glycolysis; ACC = absolute correlation coefficient; BLR = bone marrow-to-liver ratio ; SLR = spleen-to-liver ratio ; TBR = tumor-to-background ratio ; TMR = tumor-to-muscle ratio ; OS = overall survival ; PFS = progression free survival

An experienced nuclear medicine physician analyzed and segmented the FDG-PET/CT scans without knowledge of the patients’ clinical findings, as in the study by Iravani et al. [59]. This is a bicentric study, which is not the case with the previously cited studies, giving it a certain strength, but it is a retrospective study on a relatively small number of patients, which introduces a selection bias. Nevertheless, the population remains homogeneous, as it is a wild-type BRAF population that has only received anti-PD1 as first-line treatment exclusively. Furthermore, this is the only report to have evaluated TFs Finally, as with all the studies cited in this chapter, there was no validation on an external cohort.

## Predictive mutational status approach

Survival of patients with advanced CM is still limited, reaching barely 25–30% at 5 years [62]. However, this survival could be significantly improved by targeted therapies, which offer 3-year survival rates of 37%, depending mainly on genomic status [63], with BRAF- (50%), NRAS- (15–20%) and proto-oncogene (c-Kit) (2–3%) mutations being the most common [64]. However, these individualized therapies rely on genomic analysis of tumor cells, which requires prior biopsy and histopathological evaluation. Any imaging technique that provides information on mutational status could be of considerable benefit, avoiding time-consuming histopathological analysis and allowing for earlier initiation of targeted therapy, thereby optimizing outcomes. Few series have been reported on this subject in literature.

Saadani et al. [65] conducted the first study on a cohort of 70 patients with unresectable stage III-IV melanoma to predict BRAFV600 mutation status using selected radiomic TFs derived from FDG PET/CT with molecular biology as the gold standard. Patients were divided into 2 groups according to their mutation status: BRAFV600 group (35 patients, 100 lesions) and wild-type BRAF group (35 patients, 79 lesions). The size of the tumor lesion was measured in the axial plane. Measurable disease was defined as lesions of at least 2 cm or (if the tumor was indistinguishable on CT) by an equivalent MTV of at least 4.2 ml. from each patient’s measurable lesions, the 3 lesions with the highest SUVmax per organ were considered target lesions. Target lesions were delineated by a relative threshold of SUVmax > 50% without background correction. The specified organ regions were consistent with the melanoma metastasis model: lymph nodes, lung, liver, bone, subcutaneous, intramuscular, and others. For radiomic analysis, 485 features were extracted, including 5 conventional PET parameters (SUVmax, SUVmean, SUVpeak, MTV and TLG) and others related to morphology (*n* = 22), local intensity (*n* = 2), intensity-based statistics (*n* = 18), intensity-volume histogram (*n* = 6), intensity histogram (*n* = 24) and texture (*n* = 408). TFs were based on GLCM, GLRLM, GLSZM, gray level distance zone matrix (GLDZM), the neighborhood gray tone difference matrix (NGTDM) and NGLDM. For feature selection, the authors considered 6 different approaches, which they cross-validated 10 times. For each patient, 1 to 10 target lesions were analyzed. The BRAFV600 and wild-type BRAF groups were not statistically different with respect to SUV, MTV, TLG, longest diameter, or prior local intervention metrics. Stratification by organ region for SUV and TLG metrics yielded the same result. The best prediction model based on conventional PET features included all of them, i.e., SUVmean, SUVmax, SUVpeak, TLG, and MTV. Texture analysis was performed on 176 lesions (3 lesions from scans with a different voxel matrix were excluded). AUCs predictive of BRAFV600 mutation ranged from 0.54 to 0.62. The authors conclude that BRAFV600 mutation status was neither associated with nor predicted by conventional PET features, whereas TFs had predictive value (AUC of 0.54 to 0.62). Patients were scanned on a Phillips Gemini TF 16 or Phillips Gemini TF big-bore FDG-PET/CT scanner, cross-calibrated (with calibration phantoms) for 1–3 min per bed position. The systems were from the same supplier and had the same type of image acquisition and reconstruction methods, and the same settings were used. This guarantees the quality of the image analysis. However, the methods used to analyze the FDG-PET/CT images were not specified, which is a methodological shortcoming. Regarding the population, it was derived from a monocentric retrospective collection, which is synonymous with selection bias. However, even if the number of patients is small, this population remains homogeneous. Finally, in the absence of an external validation cohort, the authors carried out 10 cross-validations, which could lead to highly biased results if the TFs are poorly applied [66].

Furthermore, Olthof et al. [67] evaluated the potential of CT texture analysis parameters and metabolic characteristics of melanoma metastases on FDG-PET/CT to predict relevant mutations of tumor cells prior to targeted therapy in correlation with histopathological specimen. Sixty-six patients undergoing contrast-enhanced FDG-PET/CT before planned metastasectomy without any prior systemic therapy were included in this single-centre retrospective analysis. Both quantitative analysis on FDG-PET (including MTV, SUVmax, SUVpeak and SUVmean) and CT analysis (including attenuation Standard Deviation (SD), Kurtosis, Skewness, Mean value of Positive Pixels (MPP), Uniformity of Distribution of Positive Pixels (UPP), Entropy, Uniformity) were calculated in the largest resected metastasis in a delineated area with a relative threshold of SUVmax < 40%. Tissue samples were analysed for BRAF (*n* = 21), NRAS (*n* = 11), c-Kit (*n* = 1) and wild-type (*n* = 23). In 10 patients, including 3 patients with uveal melanoma, the mutation status was unknown. The attenuation SD within the target lesion showed a weak correlation with its SUVpeak (rho − 0.292, *P* = 0.017). However, no correlation between metabolic FDG-PET parameters and tumor cell mutation was found [68]. All patients were examined on the same whole-body FDG-PET/CT system (Biograph mCT, Siemens Healthineers, Knoxville TN, USA). This ebsures the quality of image analysis. Patients were evaluated retrospectively by two readers blinded to any clinical data using dedicated software. With regard to the population, it was derived from a monocentric retrospective collection, which is synonymous with selection bias.

## Methodological aspect

The methodological aspect of radiomic studies, including texture analysis, remains essential to ensure reproducibility of results in clinical practice [38]. Furthermore, intra-tumoral heterogeneity in the case of multimetastatic disease is one of the problems of melanoma. Because of this characteristic, manual tumor delineation is an extremely time-consuming process, which can make it difficult to use clinically in routine practice. Typically, target lesions are quantified and used for follow-up. However, more recent results suggest the use of total MTV as a single parameter or in combination with the number of new lesions to improve therapy assessment and outcome prediction [69]. Many studies have suggested that TFs are affected by region size [70, 71], which is crucial for the classification of highly heterogeneous regions of varying size. The first threshold of 64-voxel was chosen after most research papers used this volume threshold for regions to be included, as TFs below this size provide less predictive information. Therefore, the methodological aspect in radiomic studies remains essential to ensure the reproducibility of results for clinical practice [38]. Recently, a standardization of radiomic procedures has been proposed to avoid the lack of reproducibility and to allow harmonization of practice, [72, 73]. In the future, radiomics will also undoubtedly include artificial intelligence to increase its robustness, [39].

The studies mentioned above in this review [60, 65, 67, 74] used different thresholding techniques for image segmentation. The most commonly used absolute threshold of SUV = 2.5 produces many false positive regions of interest, initially overestimating tumor regions and making manual correction very laborious and time consuming. Nevertheless, a relative threshold of 40%SUVmax has been reported as a more reproducible method of tumor delineation and the best approximation for estimating tumor volume [75]. Indeed, Guezennec et al. demonstrated high interobserver reproducibility with this method for calculating TFs in an analysis of 43 head and neck cancers, showing excellent agreement for 3 indices (GLCM Homogeneity, GLCM Correlation, and GLCM Entropy) with an intraclass correlation coefficient greater than 0.90 [76]. In practice, thresholding methods offer the following advantages: simple, fast calculation, good results on images with objects having uniform intensity values against a high-contrast background. They also work well on images with variable backgrounds and very small, sparse objects. However, these methods have a number of disadvantages, as they give poor results on images with low object/background contrast, generate more noise, a background intensity with significant variation across the image/high illumination gradient, and are quite computationally expensive [77]. Another commonly used segmentation technique is based on the PERCIST approach for treatment response assessment in delineating the target tumor with a threshold of 1.5 x mean liver SUV in a 3-cm spherical VOI in the right lobe + 2 SD [58, 59]. This technique has the same limitations as absolute SUV-based thresholding, namely the difficulty in dealing with intratumoral heterogeneity. In addition, the gradient-based method allows assessment of tumor in easily excluding areas of necrosis, but is not widely available, although it is highly reproducible [76, 78].

Another pitfall is the size of the tumors. The disadvantage of calculating TFs for regions larger than 64 voxels [79] is not really relevant for a disease like advanced CM, which spreads throughout the body with heterogeneous regions of different sizes, even smaller than a few milliliters. Nevertheless, higher order features require more space to manifest their properties. Thus, some studies suggest that these second or higher order TFs are highly dependent on volume size [70, 71, 80], as gray-scale regions are less present in small volumes. However, it has previously been shown that quantification at 64 Gy levels provides the best compromise between sufficient sampling of voxel SUVs, preservation of original intensity data and potential additional information regarding MTV [80]. This suggests that for spherical lesions, a minimum volume of at least 4 cm^3^ using a voxel size of 4 mm (4 × 4 × 4 = 64 voxels) is currently the retained size threshold [81, 82]. The effects of SUV discretization and spatial resampling were investigated by discretizing into a fixed number of bins (32, 64 or 128), over the full SUV range in each image, and either without spatial resampling (4.07 × 4.07 × 5.00 mm), or by resampling into 3 mm isotropic voxels. Over 60% (60.3%) of the concordance correlation coefficient (CCC) values for TFs varied by less than 5% between bin number groups. The repeatability of GLCM features was largely insensitive to changes in SUV discretization, with 80.2% of CCC values varying by less than 5% within SUV bin groups. GLZLM features were significantly affected by changes in SUV bin number, with 69.3% of CCC values varying by 5% or more (and up to 228.6%) within SUV bin groups [83]. To study the impact of harmonized image reconstructions on feature consistency, the authors performed discretization using a fixed bin number (FBN) of 64 and a fixed bin width (FBW) of 0.25. They found that the effect of image discretization resulted in better repeatability and less sensitivity to boundary differences for the FBW discretization [84]. In addition, Orlhac et al. showed that discretization with FBW resulted in more meaningful features, i.e. features capable of distinguishing tumor types well [85].

To address these specific problems in metastatic melanoma, Vagenas et al. [86] proposed a whole-body segmentation model capable of efficiently identifying highly heterogeneous tumor lesions in metastatic melanoma from whole-body 3D FDG-PET/CT images. The decision support system provides unsupervised segmentation of high FDG uptake regions based on fuzzy C-means and a custom region growth algorithm to overcome size contraints. Subsequently, a region classification model based on radiomic features and neural networks was used to classify these regions as tumors or not. The experimental results showed high performance in identifying metastatic melanoma lesions with a sensitivity of 83.7%, specificity of 91.8% and accuracy of 87.75% with an AUC of 94.2%.

Another problem with radiomics is the difference in significant TFs from study to study, probably due to the “rebound beta”, which would result from a high correlation between the plethora of TFs often analyzed. To minimize this problem, Deleu et al. [87] used the principal component analysis (PCA) technique on 123 melanoma lesions from 26 patients. This technique, which reduces the dimensionality of large datasets containing highly correlated variables, such as texture feature datasets derived from FDG-PET images, improves the interpretability of the data while minimizing the loss of information by creating new uncorrelated variables that successively maximize variance. Thirty-eight TFs were extracted from different VOIs, including GLCM, NGLDM, GLRLM and GLSZM. Using Principal Component Analysis, their dataset of 38 generated TFs could be compressed to a dataset of 5 new uncorrelated variables or PCA that explained approximately 82% of the total variance. Although this study was conducted on a small population and retrospectively, it allowed the identification of 5 new uncorrelated variables that provide summarized information on the locoregional FDG distribution with an emphasis on both high and low FDG uptake regions, contrast in FDG uptake values (steepness), tumor volume, locoregional tracer distribution and on the speed of change of SUV intensity between different regions.

The analysis of radiomic features has shown particular promise in cancer research. However, traditional radiomic feature analysis has had limited utility for patients with metastatic or multifocal disease because there are few established methods for aggregating radiomic features from multiple tumors to produce patient-level outcome estimates. Chang et al. [88] compared different radiomic feature aggregation methods and tested their performance in different survival models in a population of patients with secondary brain metastases from various solid tumors (non-small cell lung cancer (NSCLC), small cell lung cancer (SCLC), breast, melanoma, renal, gastrointestinal). The following aggregation methods were compared to estimate patient-level risk for patients with multiple metastases: (1) Unweighted average: radiomic size and shape characteristics were summed, and all other characteristics were averaged for each patient. (2) Weighted average: radiomic size and shape characteristics were summed, while all other characteristics were averaged for each patient based on a weighted proportion of the total volume of all metastases per patient. (3) Weighted average of the three largest metastases: the radiomic characteristics of size and shape of the three largest metastases in each patient were summed, while all other characteristics of the three largest metastases were averaged based on a weighted proportion of the total volume of the three largest metastases. The three largest metastases were chosen because Graded Prognostic Assessment, a widely accepted prognostic model, uses a threshold of three metastases for prognostic risk stratification. (4) Largest metastasis + number of metastases: the characteristics of each patient’s largest metastasis were selected, and the total number of metastases for each patient was included as an additional variable. (5) Largest metastasis alone. (6) Smallest metastasis alone as a control with the hypothesis that the smallest tumor would have a reduced prognostic value compared to larger tumors. The results show that a volume-weighted average of the radiomic characteristics of the three largest brain metastases is the most effective technique for modelling survival across different survival analysis methods. It also suggests that in patients with multifocal disease, the largest tumors may influence prognosis. The application of this aggregation method was used in this study, which evaluated whether the combination of radiomic and clinical parameters was superior to clinical parameters alone in predicting therapeutic response at 3 months and OS at 6 and 12 months, in 261 patients with stage IV malignant melanoma undergoing immunotherapy with PD-1 and CTLA-4 checkpoint inhibitors. Radiomic assessment was performed using CT. For each segmented lesion, 14 radiomic shape features, 18 first-order statistical features and 75 TFs were extracted. A total of 1316 features were extracted per lesion. The features were not harmonized to account for the different scanner types, each radiomic feature at the lesion level was aggregated per patient in four different ways: (1) feature value of the most important lesion; (2) volume-weighted average of the feature values of the three most important lesions; (3) feature value of the most predictive lesion, and (4) volume-weighted average of the feature values of the three most predictive lesions. Although the study was not statistically significant (*P* = 0.08) and the resulting AUCs were low (AUC < 0.7), it has the merit of following a whole-body segmentation approach of all visible metastases in order to obtain as much information as possible [89].

Finally, radiomics suffers from the problem of reproducibility. Indeed, model stability and reproducibility clearly need to be assessed before applying a predictive model in a clinical context. Indeed, it is well known that model fit is optimal in the training set used to build the model, whereas validation in an external cohort provides more reliable fit estimates. The first step in model validation is internal cross-validation. However, the best way to assess the potential clinical value of a model is to validate it with independent, prospectively collected cohorts, ideally in clinical trials. This raises the problem of data sharing between different institutions, requiring to use of shared databases as validation sets [42, 49]. One approach to solving this problem is the development of large, publicly accessible databases, such as The Cancer Imaging Archive (TCIA) [90], a public resource that hosts patient imaging data from The Cancer Genome Atlas (TCGA) database, which contains comprehensive multidimensional genomic data and clinical annotations for over 30 cancer types [91]. These images can be used as a valuable resource to generate and validate hypotheses.

## Practice harmonization strategies

The field of radiomics is expanding and offers real hope for prediction in the field of cancer. However, studies are mainly carried out in monocentric settings, with the problem of reproducibility. Indeed, the variability of radiomic steps can affect the absolute values and statistical distributions of radiomic features, and thus the interchangeability of radiomic features [36, 92]. Radiomics is a rapidly expanding field of research [93], but to date the vast majority of studies have been conducted out in a single institution, creating a “batch effect”, which is characterized by the disparity between the biological [40]. This “batch effect” is conceptually similar to variations induced in radiomic features induced by the medical imaging model, acquisition protocol and/or reconstruction settings, sometimes referred to as the “center effect”.

Various techniques for eliminating “batch effects” have been widely used when combining data from different experiments consisting of two or more data sets. The “batch effect” removal methods can be classified into three groups: matrix factorization (MF) methods, discretization methods and location-scale (LS) methods [94].

Matrix factorization methods (MF) assume that the variation in the data corresponding to batch effects is independent of the variation corresponding to the biological variable of interest and can be captured in a small set of factors that can be estimated by matrix factorization methods.

Discretization methods transform expression data into persistently defined classes based on their expression levels. This merging method can be done trivially by concatenating discretized matrices. discretized matrices. Some loss of information during discretization is inavoidable. However, it has been shown that these strategies can occasionally lead to comparable or improved accuracy, depending on the type of downstream analysis.

Location-Scale (LS) methods transform the data of each batch in order to obtain an equal mean and/or variance. Among these LS methods, there is the ComBat method, which is widely used in pathological imaging [95, 96]. ComBat identifies a lot-specific transformation to express all data in a common space without center effects. The ComBat algorithm follows a three-step procedure: (i) Data standardization, (ii) Empirical Bayes estimation of prior distribution hyperparameters from the standardized data and subsequent estimation of batch effect parameters, which are used in (iii) Batch correction [97]. It eliminates batch effects mainly on the basis of an empirical Bayes framework. It has been shown to be robust with smaller sample sizes and remains a widely used approach. When compared with five other popular “batch effect” removal methods, ComBat was found to be “most capable of reducing and eliminating batch effects while increasing precision and accuracy”. In the context of harmonizing radiomics characteristics, ComBat works by normalizing them. It has several interesting properties for radiomics harmonization as it does not require feature definitions to be modified, so it can be used with any algorithm. It’s fast and easy to use. It makes use of all available information, as none of the features are eliminated before analysis. It is machine-specific, based solely on patient data acquired in the different centers, and does not require phantom experience and it can be used for prospective or retrospective data, provided that the different centers have the same cohorts of patients with the same disease who have received the corresponding treatment. One of the limitations is that it centers the data on the overall mean of all samples, resulting in an adjusted data matrix that is shifted to an arbitrary location that no longer coincides with the location of any of the original centers. This can cause harmonized features to lose their original physical meaning (and impossible values, such as negative volumes or SUVs) [61].

A modified version of ComBat, called M-ComBat [98], centers the data on the location and scale of a predetermined “reference” batch, providing the ability to harmonize the feature set with a chosen reference. Da-Ano proposed a parametric bootstrap addition for the parameters in the ComBat and M-ComBat models, respectively [61]. The proposed use of bootstrapping for initial estimates reduced the variances within each center and facilitated bias reduction in the estimation of center effect parameters by ComBat and M-ComBat, respectively, resulting in improved center effect elimination and predictive performance. Although relatively small, the improvement from the use of Da-Ano M-ComBat was consistent.

In total, these studies were all retrospective and essentially monocentric, with the exception of the Flaus study, which involved two centers. This makes them less robust.

The overall mean number of patients in the studies reviewed was 77, with 4 studies having numbers of around 50 patients, which is relatively small for a radiomics study design. The studies [50, 54–56, 59, 60] studies used different machines with hamonization procedures to ensure the quality of analysis. Flaus et al [60], on the other hand, used the ComBat technique, which serves as a reference technique to overcome the biases introduced by different machines.

Only two studies considered TFs [60, 65].

It should also be noted that no external validation was performed. However, a cross-validation was carried out by Saadani’s team [65], but this was less robust than an external validation.

## Conclusion

Radiomics holds promise for assessing melanoma and predicting response to TKIs and ICI immunotherapy, and also has the potential to predict mutational status. but certain limitations need to be overcome. Firstly, study design must be based on a methodology with specific criteria, which would harmonize practices and promote reproducibility. Secondly, it would be essential to design prospective studies with a large number of patients, in order to overcome the heterogeneity generated by retrospective studies. This would make it possible to include in clinical practice a simple tool capable of correctly analyzing images and aiding therapeutic decision-making.

## Data Availability

Not applicable.
